# Low density of CD3+, CD4+ and CD8+ cells is associated with increased risk of relapse in squamous cell cervical cancer

**DOI:** 10.1038/sj.bjc.6604001

**Published:** 2007-10-16

**Authors:** B S Nedergaard, M Ladekarl, H F Thomsen, J R Nyengaard, K Nielsen

**Affiliations:** 1 Department of Oncology, Aalborg Hospital, Aarhus University Hospital, Denmark; 2 Department of Oncology, Aarhus University Hospital, Denmark; 3 Department of Clinical Epidemiology, Aarhus University Hospital, Denmark; 4 Stereology and Electron Microscopy Laboratory and MIND Center, University of Aarhus, Denmark; 5 Department of Pathology, Aalborg Hospital, Aarhus University Hospital, Denmark

**Keywords:** tumour infiltrating lymphocytes, stereology, immunohistochemistry, cervix carcinoma

## Abstract

The purpose of this study was to investigate the prognostic value of the primary *in situ* cellular immune response in cervical squamous cell carcinoma. A study of 102 women treated for stage IB and IIA disease, between 1990 and 2000, was performed. Paraffin-embedded cervical tissue processed at the time of diagnosis was immunostained for CD3+ (T cells), CD4+ (T helper/regulatory T cells) and CD8+ (cytotoxic T cells) cells. Immune cell profile densities were estimated using stereology. Both intra- and peritumoural cell densities were estimated. Using Cox's proportional hazards regression modelling we found an increase in cell density to decrease the risk of relapse for all three cell types. The density of peritumoural CD3+ cells seems to have the strongest potential for predicting relapse. An increase in CD3+ cell density from 795 to 2043 cells per mm^2^ (25–75 percentile) reduced the hazard ratio to 0.27.

Cervical cancer is a common cancer in women worldwide (Parkin *et al*, 2005). A necessary condition for development of cervical cancer is persistent infection with human papilloma virus (HPV) of certain subtypes (Bosch and Munoz, 2002), particularly types 16 and 18. New and promising vaccines to prevent HPV infection have been developed (Koutsky and Harper, 2006); however, the vaccines do not protect against all HPV subtypes and they primarily elicit their effect in women not already infected. Cervical cancer will, for decades to come, still remain a serious health problem, and to improve survival new parameters for prognosis and new adjuvant treatments are needed.

There are several well-known prognostic factors, including clinical stage, spread of the cancer to lymph nodes, tumour size and depth of invasion (Kristensen *et al*, 1999; Singh and Arif, 2004). Because cervical cancer is preconditioned by a virus infection, the immune response towards the cancer is potentially important for overcoming the disease by clearing or controlling the viral infection. Previous studies have shown infiltration of immune cells also to be associated with improved clinical outcome in cancer patients (Bethwaite *et al*, 1996; Chao *et al*, 1999).

Also in other cancers such as ovarian cancer (Zhang *et al*, 2003; Sato *et al*, 2005) and endometrial cancer (Kondratiev *et al*, 2004), has the immune response towards the cancer cells been associated with prognosis.

In a previous study we found a striking difference in the numbers of both intra- and peritumoural CD3+, CD4+ and CD8+ cells in biopsies between women who were cured by primary treatment for stage IB cervical squamous cell carcinomas and women who experienced a relapse. To further explore the value of these results we made this cohort study including 102 patients treated for stage IB and IIA disease at a single institution. The *in situ* cellular immune response was investigated with respect to densities of T cells (CD3+), T helper cells/regulatory T cells (CD4+) and cytotoxic T cells (CD8+) in intra- and peritumoural tissues.

## MATERIALS AND METHODS

### Patients and specimens

The study included 102 patients treated for cervical squamous cell carcinoma, International Federation of Obstetrics and Gynecology (FIGO) stage IB and IIA, at Aalborg Hospital from 1990 to 2000 (Table 1). Patients were clinically staged according to the FIGO criteria (Benedet *et al*, 2000). If operable, patients underwent hysterectomy with pelvic lymph node dissection and in cases of increased risk of relapse (assessed from depth of invasion, vascular invasion, spread to lymph nodes, invasion of the parametrium and cancer close to resection margins) radiotherapy was added. Inoperable patients were treated by radiotherapy (both external radiotherapy and brachytherapy). Patients were not included in the study if they were more than 70 years old at the time of diagnosis, or if they had previous malignant diseases. Retrospectively, paraffin-embedded cervical tissue processed at the time of diagnosis was retrieved from the files at the Institute of Pathology, Aalborg Hospital, Aarhus University Hospital, Denmark. From each tissue biopsy, one representative block containing cancer was included.

### Immunohistochemistry

New 4 *μ*m thick sections were cut from each selected block of formalin-fixed, paraffin-embedded tissue, using a calibrated rotatory microtome (Leica RM 2165). Sections were mounted on SuperFrost^R^Plus slides, dried at room temperature overnight and afterwards dried for 60 min at 60°C. The sections were deparaffinised using Tissue-Clear (Sakura, Zocterwoude, The Netherlands) for 10 min. Endogenous peroxidase blocking (3 ml 30% H_2_O_2_ in 200 ml 99% ethanol for 15 min) followed by rehydration in a series of graded alcohol (96 and 70%) and finally water was performed. Antigens were retrieved by pressure cooker (heating to 120°C in Tris/EDTA retrieval buffer). After cooling in water, slides were placed in TBS-Tween buffer for 5 min. Staining was performed using Dako autostainer at temperature 18–22°C. The primary antibodies used were mouse monoclonal anti-human CD3 (code no. M 7254, Dako, Glostrup, Denmark) 1 : 40, anti-CD4 (code no. NCL-CD4-1F6, Novocastra, Newcastle, UK) 1 : 25 and anti-CD8 (code no. M 7103, Dako) 1 : 250.

Primary antibodies were incubated for 30 min and thereafter rinsed with TBS-Tween buffer followed by amplification by a labelled polymer conjugate (Dako EnVision+kit K4007). After rinsing with TBS-Tween buffer, antigen-bound antibodies were visualised with DAB+ (K4007, Dako). After another round of rinsing with TBS-Tween buffer, the sections were counterstained with Mayers haematoxylin for 5 min. To enhance the visualisation of the staining, the sections were incubated in 0.5% CuSO_4_ in TBS buffer for 5 min. At last the sections were mounted with Pertex.

Tonsil tissue was used as positive control and it was stained together with the sections included in the research study.

### Evaluation of immunostains

An Olympus light microscope (BH-50) equipped with a video camera and connected to a PC with a computer-assisted stereology system (CAST version 2, Olympus, Ballerup, Denmark) was used for evaluation of the immunostained sections. The microscope had a motorised stage controlled by the computer for precise and random selection of fields of view within the entire area of cancer. Immune cells were counted with respect to their location, that is, intra- or peritumoural. An area containing stromal tissue was denoted peritumoural, if at least one malignant cell was observed in the field of view inside a sampling frame of an area of 31 × 10^3^ *μ*m^2^ as described in detail previously (Nedergaard *et al* in press).

The chosen density of fields of view varied depending on the density of the cells of specific subtypes to be assessed, as we aimed to count at least 50 cells of each subtype. Cell profiles were counted at a final screen magnification of × 1024 using a 2D counting frame and a simple, unbiased counting rule (Gundersen, 1977). For each of the three cell immunotypes, the number of cell profiles and their anatomical position (intra- or peritumoural) were registered.

## STATISTIC ANALYSIS

Cox's proportional hazards regression modelling was applied to estimate the hazard rates. Both multiple and univariate models for the different immune subtypes were performed. Crude and adjusted hazard ratios were calculated. The models were adjusted for age at diagnosis and FIGO stage. Based on the number of patients it seemed unreasonable to adjust for more variables. Kaplan–Meier survival curves were drawn. For analysis the statistical software package R version 2.4.1 was used.

## RESULTS

In Table 2 the densities of cell profiles are divided into quartiles. The fraction of patients having a relapse is shown for each group. Generally, for all three cell subtypes the lower the density of cell profiles the higher the fraction of patients having relapse. The density of peritumoural CD3+ cells seems to have the strongest potential for predicting relapse. In the group of patients having the lowest density of CD3+ cells, that is, first quartile, 62% had a relapse. In the group of patients having the highest density of CD3+ cells, that is, fourth quartile, only 12% had a relapse. In both the second and third quartile 24% had experienced a relapse.

In a multiple regression model with age at diagnosis, FIGO stage and the three immune cell subtypes, none of the immune cell subtypes were independent predictors. The hazard ratios given in Table 3 are based on univariate analyses and the adjusted hazard ratios are adjusted for age at diagnosis and FIGO stage. The estimates were based on changes in cell density from 25 to 75 percentiles. The crude and the adjusted model gave almost identical results. For all cell types the hazard ratio was less than 1, that is, the risk of relapse decreased when going from a cell density at the 25 to the 75 percentile. The largest decrease in risk of relapse was found looking at peritumoural CD3+ cells. A change in cell density from 795 to 2043 cells per mm^2^ reduced the risk of relapse almost four times.

**Figure 1:**
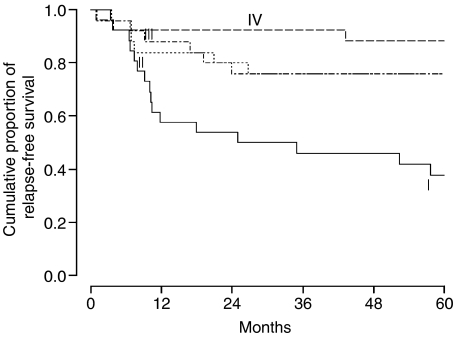
Relapse-free survival in stage IB-IIA shown for each quartile of density of peritumoural CD3+ cells.

Figure 1 shows the cumulative proportion of relapse-free survival for each quartile of peritumoural CD3+ cell density. The relapse-free survival clearly differs between the first and forth quartiles. The second and third quartiles have almost identical relapse-free survival.

## DISCUSSION

In the present study we used stereology for accurate estimation of the 2D densities of CD3+, CD4+ and CD8+ cell profiles in intra- and peritumoural tissues. We investigated the association between risk of relapse and cell densities.

In this cohort study including stage IB and IIA cervical squamous cell carcinoma, 30% of the patients had a relapse of disease. This is consistent with the findings in a study by Dupont *et al* (2005) where 22% of patients having stage IB disease and 60% having stage IIA disease experienced a relapse. The study included both squamous cell carcinomas and other subtypes.

In this study there was no or very little difference between the crude and adjusted hazard ratios for all cell types. Neither the age at the time of diagnosis nor the FIGO stage did affect the hazard ratios. However, there were only 10 patients having stage IIA disease and therefore the effect of FIGO stage on hazard ratios could not be seen. Adjustment for age was done because the ability to raise a proper immune response changes with age (Hakim *et al*, 2004); however, the age at diagnosis did not differ between groups (*P*=0.1) and no effect of age was found.

We found an increase in the density of both CD3+ and CD8+ cells to decrease the risk for relapse of disease. The decrease in hazard ratio was highly significant for both intra- and peritumoural cells. An increase in density of CD4+ cells also decreased the hazard ratio for relapse of disease, but it was only borderline significant for intratumoural cells. The largest decrease in hazard ratio was found for peritumoural CD3+ cells and it was 0.27 when increasing the cell density from 795 to 2043 cells per mm^2^.

The CD3 antigen is a protein complex comprising the T-cell receptor. The antigen is expressed on all T cells and as such a pan T-cell marker, which means that cells expressing this marker constitute a mixture of T cells including both T helper, regulatory and cytotoxic T cells. The presence of higher numbers of CD3+ tumour infiltrating lymphocytes compared to lower numbers was in a study by Bethwaite *et al* (1996) associated with improved survival in stage IB cervical cancer. Chao *et al* (1999) consistently found a pronounced lymphocyte infiltration to be an independent factor of disease-free survival in stage IIB cervical squamous cell carcinoma. Also studies of the immune response towards other tumour types have found an association between CD3+ cells and prognosis (Johnson *et al*, 2000; Zhang *et al*, 2003).

CD4 is a glycoprotein expressed on the surface of T helper cells and is a co-receptor for the T-cell receptor. The antigen is also expressed on regulatory T cells, monocytes, macrophages and dendritic cells. Because of the differences in morphology of the CD4+ cells, it is possible to distinguish between T cells and the other CD4+ cells. However, in this study we cannot distinguish between T helper and regulatory T cells. Without distinction between T helper and regulatory T cells previous studies has been able to find an association between prognosis and infiltration of CD4+ T cells (Sheu *et al*, 1999), and low CD4+/CD8+ ratio was indirectly associated with worse prognosis (Sheu *et al*, 1999). However, we did not find the ratio between CD4+ and CD8+ cells to be correlated with prognosis.

CD8 is also a glycoprotein serving as a co-receptor for the T-cell receptor. It is expressed on cytotoxic T cells that have the capability of killing tumour cells. In a recent study by Piersma *et al* (2007) the presence of intratumoural CD8+ cells in cervical cancer correlated with the lack of pelvic lymph node spread and therefore correlated indirectly with prognosis. Also in other cancer types as ovarian cancer (Sato *et al*, 2005), endometrial cancer (Kondratiev *et al*, 2004), colon cancer (Guidoboni *et al*, 2001) and lung cancer (Eerola *et al*, 2000) have intratumoural CD8+ cells that have been correlated to improved prognosis.

The immune response towards cancer is very complex and the different cell types interact and influence each other (Eiben *et al*, 2002). In our study we found CD3+ cells to be the strongest predictor of prognosis and the reason might be that the presence of several immune cell subtypes and not one in particular is important. The cytotoxic T cells are very important in cancer defence but their effect is dependent on the presence of other cells, including T helper cells. We found both intra- and peritumoural cell densities to be associated with prognosis and as opposed to other studies (Bethwaite *et al*, 1996; Chao *et al*, 1999) the densities of peritumoural CD3+ and CD8+ cells were stronger predictors than intratumoural cells.

According to our study, analyses of the *in situ* cellular immune response at the time of diagnosis can divide patients into groups, having different risks of relapse even after therapy. A relative simple evaluation of the density of peritumoural CD3+ cells is a possibly useful predictive marker of relapse, and the group of patients having the lowest density of cells should be controlled extra carefully for relapse of disease.

## Figures and Tables

**Table 1:** Patient characteristics

	**Disease free**	**Relapse**
Number of patients	71 (70%)	31 (30%)
Age at diagnosis^a^	45 [26–68]	41 [22–70]
Stage IB	64 (90%)	28 (90%)
Stage IIA	7 (10%)	3 (10%)
Primary treatment surgery	62 (87%)	26 (84%)
Adjuvant radiotherapy	13 (21%)	10 (38%)
Primary treatment radiotherapy	9 (13%)	5 (16%)

a Mean and range in brackets.

**Table 2:** Fraction of patients having relapse in each quartile of cell density

	**Quartile**	**Cells per mm^2^**	**Fraction of patients having relapse**
CD3 intratumoral	I	30–293	0.50
CD3 intratumoral	II	294–643	0.36
CD3 intratumoral	III	644–1117	0.28
CD3 intratumoral	IV	1118–3826	0.08
CD3 peritumoral	I	150–794	0.62
CD3 peritumoral	II	795–1300	0.24
CD3 peritumoral	III	1301–2042	0.24
CD3 peritumoral	IV	2043–6181	0.12
			
CD4 intratumoral	I	0–15	0.38
CD4 intratumoral	II	16–104	0.48
CD4 intratumoral	III	105–212	0.20
CD4 intratumoral	IV	213–1546	0.15
CD4 peritumoral	I	0–105	0.52
CD4 peritumoral	II	106–228	0.31
CD4 peritumoral	III	229–462	0.28
CD4 peritumoral	IV	463–2268	0.12
			
CD8 intratumoral	I	9–197	0.50
CD8 intratumoral	II	198–440	0.28
CD8 intratumoral	III	441–851	0.24
CD8 intratumoral	IV	852–5217	0.19
CD8 peritumoral	I	0–447	0.54
CD8 peritumoral	II	448–709	0.36
CD8 peritumoral	III	710–1233	0.20
CD8 peritumoral	IV	1234–4025	0.12

**Table 3:** Cox's proportional hazards model (univariate) of relapse of disease

	**Crude model**			**Adjusted model**		
	**Crude HR**	**95% CI**	***P*-value**	**Adjusted HR**	**95% CI**	***P*-value**
CD3 intratumoral 294/118	0.48	[0.27–0.86]	0.01	0.49	[0.28–0.86]	0.01
CD3 peritumoral 795/2043	0.27	[0.13–0.58]	0.0008	0.29	[0.14–0.61]	0.001
CD4 intratumoral 16/213	0.55	[0.31–1.00]	0.05	0.55	[0.31–1.00]	0.05
CD4 peritumoral 106/463	0.62	[0.39–0.99]	0.04	0.61	[0.38–0.98]	0.04
CD8 intratumoral 198/852	0.41	[0.21–0.81]	0.01	0.41	[0.21–0.80]	0.009
CD8 peritumoral 448/1234	0.38	[0.20–0.74]	0.004	0.37	[0.19–0.73]	0.004

HR=hazard ratio.

Cox's proportional hazards regression modeling was performed to determine the association between density of immune cells and relapse of disease. In the model the cell density at the 25 and 75% percentiles was compared, densities shown for each cell type. The model was adjusted for age at diagnosis and FIGO stage.
